# Erythema Migrans Caused by *Borrelia spielmanii*, France

**DOI:** 10.3201/eid2911.230149

**Published:** 2023-11

**Authors:** Pascal del Giudice, Fabienne Freychet, Lora Kopec, Florence Fenollar, Carole Eldin, Marine Velin, Thomas Hubiche, Didier Raoult, Oleg Mediannikov

**Affiliations:** Dermatology Infectiology Unit, CH Fréjus-Saint-Raphaël, France (P. del Giudice, M. Velin, T. Hubiche);; Dermatology private practice, Nice, France (F. Freychet);; MEPHI, IHU Méditerranée infection, IRD, Aix Marseille Université, Marseille, France (L. Kopec, F. Fenollar, C. Eldin, D. Raoult, O. Mediannikov);; VITROME, IHU Méditerranée infection, IRD, Aix Marseille Université, Marseille (L. Kopec, F. Fenollar, C. Eldin, D. Raoult, O. Mediannikov)

**Keywords:** erythema migrans, *Borrelia spielmanii*, bacteria, Lyme borreliosis, Lyme disease, tick-borne infections, vector-borne infections, rash, France

## Abstract

We describe a rare case of early Lyme borreliosis in France caused by *Borrelia spielmanii*, which manifested as a large erythema chronicum migrans rash. The patient completely recovered after a 15-day course of amoxicillin. Absence of pathognomonic signs prevented distinguishing *B. spielmanii* from other etiologies as cause in this case-patient.

The causative agents of borreliosis, also known as Lyme disease, in Europe are *Borrelia garinii*, *B. afzelii*, and, more rarely, *B. burgdorferi* sensu stricto. Lyme borreliosis is endemic in France except the southern region. Mean annual incidence of Lyme borreliosis in France may be as high as 84 cases/100,000 persons (*1*). 

*B. spielmanii* is a rare agent of Lyme borreliosis first isolated in 1993 from a patient with erythema chronicum migrans (ECM) in the Netherlands (*2*), then well-characterized as a novel genetic variant in 1999 (*3*). *B. spielmanii* was described as a new species in 2006 with a type strain isolated from *Ixodes ricinus* ticks collected from a garden dormouse in the Petite Camargue Alsacienne region in France (*4*,*5*). A few human cases were reported from Germany (*4*), Hungary (*6*), Czech Republic (*7*), Denmark, and Slovenia (*8*); patients in all reported cases exhibited ECM (*9*). *B. spielmanii* has been found in a low percentage of ticks feeding on dogs in the United Kingdom, Switzerland, and Belgium and on birds in Poland. Also, ticks in Austria, Denmark, and the Czech Republic (some removed from humans), *I. ricinus* ticks in the Crimea peninsula (*10*), as well as in animal tissues from Poland (red fox) (*11*) and Hungary (hedgehogs), have been shown to carry *B. spielmanii* (*12*). No infected humans have been identified France. 

## The Study

**Figure 1:**
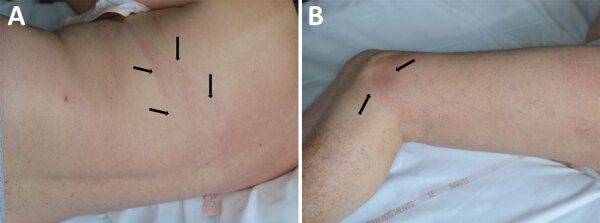
Large erythema chronicum migrans rash on a 60-year-old woman in France that was later determined to be caused by *Borrelia spielmanii*. A) Edges of linear erythema band on patient's back, indicated by arrows. B) End of linear erythema band beginning on patient's back, indicated by arrows on patient's knee.

A 60-year-old woman sought treatment on November 15, 2017, for 2 erythematous linear asymptomatic and noninfiltrated bands on her left knee and the left side of her thorax. In August 2017, the woman had noticed an annular erythema initially in the middle and on the left side of her back. The annular erythema had gradually extended to the upper left side of her back to form a single erythematous band (Figure 1, panel A) and progressed to the left knee, also forming an erythematous band (Figure 1, panel B). Both general and skin exams were otherwise unremarkable. We hypothesized that the rash might constitute an unusually large ECM. Because Lyme borreliosis is unknown in that area of southeastern France, we questioned her about her recent travel history. She had spent the week of June 14–20, 2017, in the county of Oise, north of Paris, where Lyme borreliosis is endemic. We performed a punch biopsy on the thoracic erythema band and prescribed oral amoxicillin (1 g 3×/d for 15 d), which resolved the ECM. 

We screened a serum sample from the patient using an enzyme-linked immunoassay (Liaison *Borrelia burgdorferi*; DiaSorin, https://www.diasorin.com), which revealed presence of IgG and absence of IgM for *B. burgdorferi* sensu lato. Western blotting using LymeCheck Optima IgG & IgM (Biosynex, https://www.biosynex.com) revealed presence of *Borrelia* spp.–specific IgG for p100, VlsE, p58, and p41 antigens and a faint band for *B. spielmanii*–specific *ospC* antigen (Appendix Figure). Two weak bands showed IgM for *B. spielmanii* p100 and p41 antigens (Appendix Figure). 

**Figure 2:**
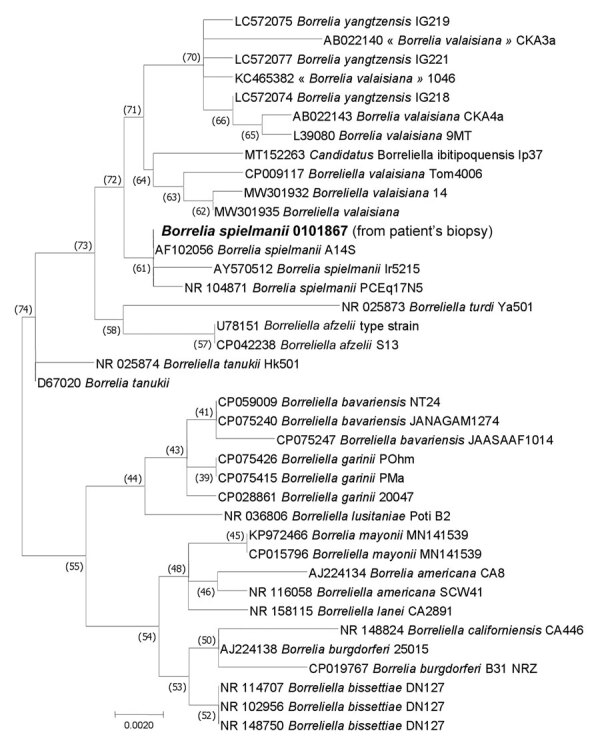
Maximum-likelihood phylogenetic tree of the 16s rRNA gene (rrs) of *Borrelia* genus bacteria showing the position of the *B. spielmanii* sequence obtained from the patient (large bold font) Evolutionary analyses were conducted using TOPALi version 2.5 (http://www.topali.org). The sequences of the 16S rDNA amplified in this study with other 12S rDNA tick sequences available on GenBank (910 positions in the final dataset) were aligned using ClustalW (https://www.genome.jp/tools-bin/clustalw) implemented on BioEdit version 3 (https://bioedit.software.informer.com). The evolutionary history was inferred by using the maximum likelihood method based on the Hasegawa–Kishino–Yano model plus invariate sites plus gamma distribution. The percentage of trees in which the associated taxa clustered together is shown next to the branches. GenBank accession numbers are provided. Scale bar indicates nucleotide sequence divergence.

We amplified portions of 16S rRNA (*13*) and *ospA* (*5*) borrelial genes from the biopsy sample. BLAST search (https://blast.ncbi.nlm.nih.gov/Blast.cgi) of sequenced amplicons showed 100% identity of the biopsy sequences with strain A14S of *B. spielmanii* for both the 910 bp–long portion of 16S rRNA gene (Genbank accession no. AF102056) and 260 bps-long amplicon of *ospA* (Genbank accession no. CP001469). We deposited our sequences into Genbank (accession nos. OR192893 and OR234396). The phylogenic tree (Figure 2) showed that the sequence obtained from the patient clusters with other *B. spielmanii* strains*.*

Previously, only *B. azfelii*, *B. garinii*, and *B. burgdoferi* sensu stricto had been identified from patients in France (*14*). Our patient manifested a rare clinical form of ECM, with a large erythema migrans across her body, extending from her upper back to her knee. However, the unusually large size might have resulted from delays in seeking treatment and diagnosis, so that particular clinical manifestation might not be specific to *B. spielmanii*. 

We reviewed available literature on clinical descriptions of human cases of *B. spielmanii* infection and found only 2 published case reports. A 69-year-old woman from Slovenia showed skin manifestations described as redness, mild local itching, burning, and pain on the left knee and later a 24 × 20 cm ring-like lesion on the left thigh (*8*), but she had no identified tick bite. In a second case, a 42-year-old woman from Hungary exhibited an ECM 10 cm in diameter on her knee (*6*). Clinical manifestations were missing in other case reports (*3*,*9*), in which only human isolates were described. In 1 study, *B. spielmanii* was detected in isolates from 4/242 patients with ECM from Germany and Slovenia (*9*). However, in that study, 3 of the 4 patients infected with *B. spielmanii* lived in Munich where a higher proportion of ticks were positive for that pathogen. This finding argues for sporadic occurrences of the infection in other locations. 

In summary, our study has contributed more data on *Borrelia* spp. as potential causes of Lyme disease, prompting need for broader surveillance. However, additional well-documented reports on ECM as a manifestation of *B. spielmanii* are needed to provide new information about the epidemiology of this *Borrelia* in Europe. 

Appendix Additional information on study on Lyme disease caused by *Borrelia spielmanii* in France.
