# Supplementary material for: Erythema Migrans Caused by Borrelia spielmanii, France
Source: Emerg Infect Dis. 2023 Nov;29(11):2366–9. doi: 10.3201/eid2911.230149 (PMC10617338; doi:10.3201/eid2911.230149)
Supplement: Appendix — Additional information on study on Lyme disease caused by Borrelia spielmanii in France. [file 23-0149-Techapp-s1.pdf]

Article DOI: https://doi.org/10.3201/eid2911.230149

Erythema Migrans Caused by *Borrelia spielmanii*, France

Appendix

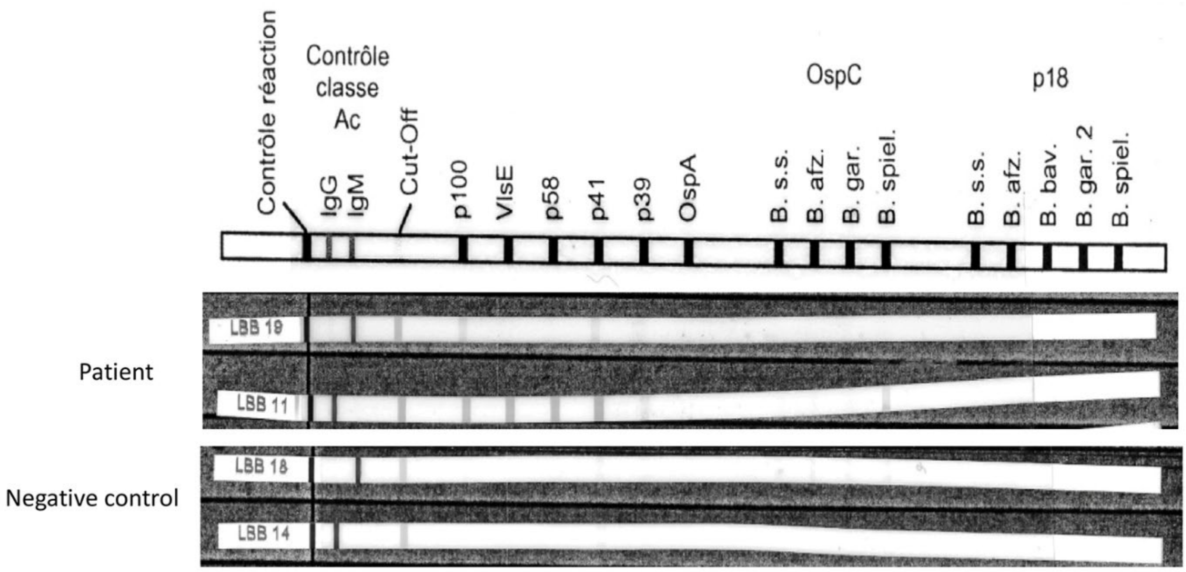

Appendix Figure. IgM and IgG results from a western blot test of serum from the patient and a negative control. Ac, activator; B, *Borrelia*; B. afz, *Borrelia afzelii*; B. bav, *Borrelia bavariensis*; B. gar, *Borrelia garinii*; B. s.s., *Borrelia* sensu stricto; osp, outer surface protein; vis, visium spatial gene
